# Cardiovascular Autonomic Dysfunction in Patients of Nonalcoholic Fatty Liver Disease

**DOI:** 10.1155/2016/5160754

**Published:** 2016-12-08

**Authors:** Mavidi Sunil Kumar, Akanksha Singh, Ashok Kumar Jaryal, Piyush Ranjan, K. K. Deepak, Sanjay Sharma, R. Lakshmy, R. M. Pandey, Naval K. Vikram

**Affiliations:** ^1^Department of Internal Medicine, All India Institute of Medical Sciences, New Delhi 110029, India; ^2^Department of Physiology, All India Institute of Medical Sciences, New Delhi 110029, India; ^3^Department of Radiodiagnosis, All India Institute of Medical Sciences, New Delhi 110029, India; ^4^Department of Cardiac Biochemistry, All India Institute of Medical Sciences, New Delhi 110029, India; ^5^Department of Biostatistics, All India Institute of Medical Sciences, New Delhi 110029, India

## Abstract

*Aim.* The present study was designed to evaluate the heart rate variability (HRV) in nonalcoholic fatty liver disease (NAFLD) and to assess the effect of grade of NAFLD and diabetic status on HRV.* Methods.* This cross-sectional study included 75 subjects (25 NAFLD without diabetes, 25 NAFLD with diabetes, and 25 controls). Measurements included anthropometry, body composition analysis, estimation of plasma glucose, serum lipids, hsCRP, and serum insulin. HRV analysis was performed in both time and frequency domains.* Results.* The time and frequency domain indices of overall variability (SDNN, total power) were significantly lower in NAFLD with diabetes as compared to the controls. However, the LF : HF ratio did not differ among the three groups. The variables related to obesity, lipid profile, and glucose metabolism were also higher in NAFLD with diabetes and those with Grade II NAFLD without diabetes, as compared to controls. Multivariate stepwise regression analysis showed a negative correlation between HRV and total cholesterol and fat percentage.* Conclusion.* The grade of NAFLD as well as diabetic status contributes to the decrease in the cardiovascular autonomic function, with diabetic status rather than grade of NAFLD playing a critical role. Serum lipids and adiposity may also contribute to cardiac autonomic dysfunction.

## 1. Introduction

Nonalcoholic fatty liver disease (NAFLD) is a clinicopathological condition characterised by lipid deposition in the liver and is a common cause of liver dysfunction. The prevalence of NAFLD in Asian population ranges from 9% to 45% [1] and is estimated to be about 30% in western population [2]. Apart from an increased risk of liver-related morbidity and mortality, patients of NAFLD also have higher cardiovascular risk [3, 4] especially when present along with type 2 diabetes (T2DM) [4, 5].

A balanced autonomic output to liver is important for maintenance of circadian rhythms of liver metabolic enzymes and glucose level [6]. Imbalance in autonomic function has been proposed as a component in pathogenesis of NAFLD [7]. The autonomic dysfunction has been shown to be higher in the NAFLD. The autonomic symptom burden assessed by orthostatic grading scale was higher in nondiabetic NAFLD [8] and the sudomotor dysfunction was also higher in the NAFLD after accounting for all confounders [9].

A recent systematic review of 27 studies showed an association of NAFLD with subclinical atherosclerosis independent of traditional risk factors and metabolic syndrome [10]. Similar independent association of NAFLD with subclinical atherosclerosis has been observed in Asian Indians also [11]. In other clinical settings, it has been shown that arterial stiffness is inversely related to heart rate variability [12, 13]. However, there are only a few studies in which heart rate variability has been evaluated in NAFLD [9, 14, 15]. Decrease in indices of HRV has been reported by Liu et al. with decrease in parasympathetic component after adjustment of covariates [14]. A higher LF : HF ratio (low frequency : high frequency) of HRV was reported in patients (*n* = 18) with NAFLD along with a lower baroreflex sensitivity [15].

Cardiovascular autonomic neuropathy is a common complication of diabetes [16]. NAFLD commonly coexists with diabetes. Both may act synergistically leading to varied clinical outcomes [17]. The independent contribution of diabetes and grade of NAFLD in development of autonomic dysfunction is not known. The present study was designed to evaluate cardiovascular autonomic status in the patients of NAFLD with or without diabetes as compared to control population of individuals without either diabetes or NAFLD. In the present study we evaluated the association of indices of HRV with anthropometric variables, lipid profile, and diabetic status in patients with and without NAFLD.

## 2. Methods

This was a cross-sectional study approved by the Institute Ethics Committee of the All India Institute of Medical Sciences (AIIMS), New Delhi. The subjects were recruited from the medicine outpatient department. Informed written consent was obtained after explanation of the purpose, type, and duration of the study.

### 2.1. Study Subjects

A total of 75 subjects were recruited for the study. The subjects were grouped into NAFLD patients without diabetes (*n* = 25, 13 females and 12 males), NAFLD patients with diabetes of <5 years of duration (*n* = 25, 17 females and 8 males), and healthy controls with similar age distribution (*n* = 25, 9 females and 16 males). Subjects with alcohol consumption >140 gm/week, any secondary cause of fatty liver, hereditary disorders, any severe acute or chronic illness, seropositivity for hepatitis B, hepatitis C, and HIV, established coronary heart disease, pregnancy, and lactation were excluded. Diagnosis of NAFLD was made using ultrasonography performed by an experienced sonologist who was blinded to the clinical data of the patients, using a 3.5 MHz convex transducer by subcostal and intercostal approach (Volusion, GE).

### 2.2. Measurements

#### 2.2.1. Anthropometry and Body Composition

A detailed clinical history and clinical examination was done. All the subjects underwent anthropometric measurements and body composition analysis by bioelectrical impedance method as described earlier [11]. Height and weight were recorded to the nearest 0.1 cm and nearest 0.1 kg with stadiometer and electronic scale. Body Mass Index (BMI) was calculated by weight (kg)/height (m^2^). Waist circumference (WC) was measured midway between the iliac crest and the lower most margin of the ribs. Hip circumference (HC) was measured at the maximum circumference of buttocks. Body fat and percentage body fat were estimated by foot-to-foot bioelectrical impedance technique (TANITA, Japan).

#### 2.2.2. Biochemical Measurements

Venous blood samples were obtained after an overnight fast of at least 10 hours. Biochemical measurements included liver function tests, fasting blood glucose (FBG) and postprandial blood glucose (PPBG) levels, and fasting lipid profile using standard methods as described earlier [11]. Serum levels of hsCRP were measured using a commercially available reagent kit based on the principle of solid phase enzyme-linked immune sorbent assay (Biocheck, Inc., Foster City, USA). Fasting insulin was measured using a commercially available reagent kit based on the principle of electrochemiluminescence (Liaison® Insulin, DiaSorin Inc., USA). The inter- and intra-assay coefficient of variation was <5%. The HOMA-IR was calculated by using the following formula: HOMA-IR = fasting insulin (*µ*U/mL) *∗* fasting blood glucose (mmol/L)/22.5.

#### 2.2.3. Assessment of Heart Rate Variability

The cardiovascular autonomic status was estimated in the Autonomic Function Testing Laboratory (AFT Lab), Department of Physiology, AIIMS, New Delhi. The subject was requested to lie down on the couch where electrodes for lead II ECG acquisition by Labchart Pro 7® (AD Instruments, Australia) were attached. The recording was performed in a controlled ambient temperature of 22 to 25°C. After an initial period of rest of 5 minutes, a 5-minute lead II ECG (sampling rate 1 KHz) was recorded for later offline analysis. The subject was instructed to avoid movement during data acquisition to prevent artefacts in the recording.

Offline analysis of ECG was done using Labchart Pro 7 (AD Instruments, Australia) and HRV was computed using Hemolab software (version 8.5, Harald Stauss Scientific, Iowa). For time domain analysis the beat-to-beat heart rate series was computed from ECG using Labchart Pro and was then exported as a *∗*.txt file. The file format was converted to *∗*.asc as required by the Hemolab software for batch processing. The time domain indices of HRV computed were SDNN (standard deviation of all NN intervals), SDSD (standard deviation of differences between adjacent NN intervals), and pNN50% (NN50 count divided by the total number of all NN intervals). For frequency domain analysis RR intervals were computed from ECG using Labchart Pro and were then exported as a.txt file. The file format was converted to *∗*.asc and then to *∗*.tsa for batch processing. This was followed by spline interpolation at sampling frequency of 5 Hz followed by the power spectral density (PSD) calculation using Fast Fourier Transformation (FFT). The measurement of low frequency (LF: 0.04–0.15 Hz) and high frequency (HF: 0.15–0.40 Hz) power components was made in absolute values of power (ms^2^). The powers were further normalized to account for changes in the total power of the HRV.

### 2.3. Statistical Analysis

The data was analysed using SPSS software (Version 22, IBM). The normality of distribution was estimated with Shapiro-Wilk test. Quantitative data is expressed as mean ± SD for normally distributed data, and median with interquartile range (IQR) is used to express skewed data. For parametric data, one-way ANOVA followed by Tukey's test was used and for nonparametric data Kruskal Wallis H test followed by Dunn's comparison was done. *p* < 0.05 was considered to be statistically significant. Two-way ANOVA was employed to evaluate the relative contribution of the diabetic status and grade of NAFLD. Correlation analysis was done with Spearman's test followed by stepwise multivariate analysis.

## 3. Results

The anthropometric measures, biochemical investigation, and lead II ECG recordings were measured in all the subjects. The data of 7 subjects in the NAFLD without diabetes group was excluded from HRV due to presence of artefacts that prevented computation.

### 3.1. Anthropometric Features and Biochemical Profile

Table 1 shows the general, anthropometric, and biochemical parameters of the subjects. Patients with NAFLD and diabetes were older (42.9 ± 7.6 yrs) than patients with NAFLD without diabetes (41.8 ± 7.3 yrs) and controls (37.9 ± 8.4 yrs). The BMI, fat mass, and fat percentage were higher in NAFLD with diabetes group as well as NAFLD without diabetes group as compared to the controls but there was no difference between NAFLD with and without diabetes. Value of WHR was higher only in NAFLD without diabetes as compared to the controls. The fat percentage was the only measure that was higher in the NAFLD with diabetes as compared to the NAFLD without diabetes.

The ESR was higher in NAFLD with diabetes while hsCRP was higher in NAFLD without diabetes as compared to controls. All the indices of liver function (bilirubin, SGOT, SGPT, and ALP) were not significantly different among the three groups. As expected, the values of FBG, PPBG, and HOMA-IR were significantly higher in NAFLD with diabetes group as compared to the other two groups. Serum levels of TC and LDL were higher in NAFLD with diabetes group as compared to the control group.

### 3.2. Heart Rate Variability (HRV)

The time and frequency domain indices of HRV are shown in Table 2. The overall variability (time domain: SDNN and frequency domain: total power) was lower in NAFLD with diabetes group as compared to the control group. Even though median values of the overall variability of HRV were lower in NAFLD group without diabetes as compared to controls, the difference was not statistically significant. Similarly, the overall variability of NAFLD with diabetes was not statistically different from NAFLD without diabetes even though the median values were lower. The low frequency and high frequency component of HRV and the LF : HF ratio were similar in all three groups. In time domain analysis, the indices of parasympathetic component of HRV (SDSD and pNN50) were lower in NAFLD with diabetes group as compared to controls as well as NAFLD without diabetes.

The proportional distribution of Grade I and Grade II was not different in the NAFLD groups with or without diabetes. The subjects were regrouped depending upon the grades of NAFLD irrespective of their diabetic status and statistical analysis was done (Table 3). The distribution of diabetics in NAFLD Grade I and Grade II was not statistically different. The indices of overall variability (SDNN and total power) and parasympathetic component (SDSD, pNN50) were lower in Grade II as compared to controls. Even though the median values of the same parameters were lower in Grade I when compared with controls, it was not statistically significant.

In order to delineate the relative contribution of the diabetic status and grade of NAFLD, two-way ANOVA was done on all the NAFLD subjects. The presence of diabetes was found to be significantly associated with decrease in SDSD and total power (Table 4).

### 3.3. Association between Indices of HRV and Anthropometric and Biochemical Parameters

The result of Spearman's correlation performed between the selected indices of HRV (SDNN, SDSD, and total power) and anthropometric and biochemical parameters is shown in Table 5. No significant association was found between indices of HRV, parameters of inflammation (ESR, hsCRP), liver function (serum bilirubin, SGOT, SGPT, and ALP), or insulin resistance (HOMA-IR, fasting insulin). The rest of the parameters were negatively correlated with the indices of HRV.

A further multivariate stepwise regression analysis was performed between indices of HRV as dependent variable and selected independent variables (with significant association in univariate analysis).  Table 6 shows the two models. Of all the parameters that were negatively associated with HRV, only total cholesterol (TC) and fat percentage significantly associated with SDNN and total power after multivariate analysis.

## 4. Discussion

The study was done to assess the heart rate variability in patients of NAFLD with or without diabetes. Since diabetes is known to independently cause autonomic dysfunction, the patients of nonalcoholic fatty liver disease were grouped into NAFLD without diabetes and NAFLD with diabetes.

### 4.1. Anthropometric Features and Biochemical Profile

As expected, the variables related to obesity, lipid profile, and glucose metabolism were higher in NAFLD with diabetes and those with Grade II NAFLD as compared to controls [18–20]. A higher ESR in NAFLD with diabetes and higher hsCRP in NAFLD without diabetes indicate ongoing inflammatory process. Similar high hsCRP was reported by Nigam et al. [21] and Ajmal et al. [22], even after adjusting for covariates.

### 4.2. Heart Rate Variability (HRV)

The time domain and frequency domain indices of overall variability of HRV were lower in the NAFLD with diabetes group as compared to the control. Even though the median values of these parameters were lower in NAFLD without diabetes, the difference was not significant. The indices of parasympathetic component of HRV (SDNN, pNN50) were lower in NAFLD with diabetes as compared to controls as well as NAFLD without diabetes. When the analysis was done on the basis of grades of NAFLD irrespective of the diabetic status, the indices of overall HRV were found to be lower in Grade II NAFLD as compared to controls. Though the median values of the theses indices were lower in Grade I NAFLD they were not statistically significant. This indicates an independent contribution of diabetic status as well as grade of NAFLD in development of autonomic dysfunction. Within the patients of NAFLD, the diabetic status had significant main effect in decreasing HRV rather than the grade.

Similar decrease in indices of overall HRV (Ln SDNN) and parasympathetic components (Ln RMSSD) was noted by Liu et al. [23]. In the present study, no change in normalized low frequency, high frequency, or their ratio was found. Liu et al., however, have reported lower low frequency and high frequency components of HRV [14, 23]. This difference is likely due to reporting absolute values rather than normalizing the values to total power of the HRV as was done in the present study. The data of the present study suggests that decrease in total power of HRV results from a decrease in both sympathetic and parasympathetic components. In the presence of diabetes, the decrease in the parasympathetic component is more [24]. Jakovljevic et al. had reported higher values of LF : HF ratio though it had not been compared with any control group. In the present study LF : HF ratio was similar in NAFLD with or without diabetes as compared to controls. This difference could be due to inclusion of diabetics with less 5 years of history as well as lower BMI of the study subjects in the present study as compared to the report by Jakovljevic et al. [15].

In univariate analysis, the decrease in indices of HRV was significantly and negatively correlated with variables of lipid profile, obesity, and diabetic status. Interestingly, the decrease in HRV was not associated either with HOMA-IR or with fasting insulin but with fasting blood and postprandial glucose level. These observations are in tune with the known role blood glucose level and glycation play in the pathogenesis of diabetic autonomic neuropathy [25]. However, in multivariate stepwise regression analysis, indices of HRV were negatively associated with total cholesterol and fat percentage only. The importance of total serum cholesterol in the development of diabetic autonomic neuropathy has also been reported earlier [26]. These observations are similar to a report by Pimenta et al. where the autonomic control in NAFLD (measured by heart rate recovery after maximum graded exercise test) correlated with body composition and body fat [27]. In a recent review, importance of hepatic accumulation of fat in a setting of obesity for the development of hepatic/peripheral insulin resistance has been proposed [20]. NAFLD and diabetes then independently lead to the development of cardiac autonomic dysfunction.

The observation of importance of diabetic status in decrease in overall HRV is in line with observations of increase prevalence of cardiovascular disease in NAFLD with diabetes [28–30]. A recent systematic review of 27 studies showed an association of NAFLD with subclinical atherosclerosis independent of traditional risk factors and metabolic syndrome [10]. In other clinical settings, it has been shown that arterial stiffness is inversely related to heart rate variability [12, 13]. It is probable that increase in stiffness of arteries due to subclinical atherosclerosis leads to decrease in the transducer function of the baroreceptors. A lower baroreflex sensitivity in NAFLD has been reported in a single study [15]. A decrease in baroreflex function will decrease the heart rate variability of both sympathetic and parasympathetic components.

## 5. Conclusion

The result of the present study indicates that the grade of NAFLD as well as diabetic status contributes to the decrease in the cardiovascular autonomic function with a decrease in overall variability but an unchanged sympathovagal balance. It also shows that once NAFLD is developed a further decrease is more likely due to diabetes rather than further increase in the grade of NAFLD and the important role of dyslipidemia and obesity in the cardiac autonomic dysfunction represented by heart rate variability.

## Figures and Tables

**Table 1:** General, anthropometric, and biochemical parameters of the subjects.

	Controls	NAFLD without diabetes	NAFLD with diabetes	*p* value
Age (years)	37.9 ± 8.4	41.8 ± 7.3	42.9 ± 7.6	0.04
NAFLD Grades I, II, and III (*n*)	—	15, 10, 0	16, 8, 1	0.62
*Variables related to whole body fat*				
BMI (kg/m^2^)	24.2	27.8^*∗*^	27.9^*∗*^	0.0001
	(21.7–26.6)	(24.8–31.2)	(25.7–30.1)	
WHR	0.95 ± 0.1	1.0 ± 0.1^*∗*^	1.0 ± 0.1	0.052
Fat percentage (%)	24.0 ± 6.7	26.6 ± 7.0	31.4 ± 6.2^*∗*#^	0.001
Fat mass (kg)	15.4 ± 5.6	20.2 ± 7.5^*∗*^	21.7 ± 5.2^*∗*^	0.002
*Variables related to inflammation*				
ESR (mm/hr)	12.0	18.0	20.0^*∗*^	0.020
	(8.5–19.0)	(12.0–28.5)	(10.5–33.5)	
hsCRP (mg/L)	6.7	11.3^*∗*^	8.0	0.007
	(1.5–12.3)	(9.6–13.0)	(4.0–12.0)	
*Variables related to liver function*				
Bilirubin (mg/dL)	0.6	0.5	0.5	0.916
	(0.4–0.8)	(0.4–0.8)	(0.3–0.8)	
SGOT (AST) (IU/L)	25.0	30.0	27.0	0.354
	(21.5–31.0)	(22.0–44.5)	(22.0–44.5)	
SGPT (ALT) (IU/L)	25.0	36.0	36.0	0.111
	(18.5–36.0)	(23.0–57.0)	(23.0–51.5)	
ALP (IU/L)	180.0	204.0	189.0	0.545
	(158.5–213.5)	(165.0–250.0)	(154.0–229.5)	
*Variables related to lipid profile*				
TG (mg/dL)	132.0	140.0	161.0	0.228
	(81.0–173.5)	(98.5–222.0)	(115.5–201.5)	
TC (mg/dL)	168.0	191.0	218.0^*∗*^	0.005
	(144.0–196.5)	(162.5–235.5)	(171.5–230.0)	
LDL (mg/dL)	100.0	117.0	134.00^*∗*^	0.009
	(82.5–126.0)	(99.0–138.0)	(102.0–148.5)	
HDL (mg/dL)	44.0	45.0	46.0	0.423
	(40.5–49.5)	(40.5–47.5)	(41.0–53.5)	
VLDL (mg/dL)	20.0	30.0^*∗*^	32.0^*∗*^	0.003
	(16.0–26.5)	(19.5–42.0)	(23.5–43.5)	
*Variables related to diabetic status*				
FBG (mg/dL)	89.0	97.0	118.0^*∗*#^	0.0001
	(86.0–95.0)	(90.5–101.0)	(109.5–164.0)	
PPBG (mg/dL)	119.0	129.0	179.0^*∗*#^	0.0001
	(104.0–128.0)	(118.5–142.0)	(154.5–218.5)	
Fasting insulin (*µ*IU/mL)	10.5	15.6	11.4	0.323
	(5.9–17.2)	(5.4–20.7)	(10.0–17.2)	
HOMA-IR	2.0	3.8	3.8^*∗*#^	0.0001
	(1.3–4.0)	(1.3–5.1)	(2.6–5.5)	

Values are expressed as median (IQR) or mean ± SD; _ _^*∗*^significant versus control; _ _^#^significant versus NAFLD without diabetes.

BMI: Body Mass Index; WHR: Waist Hip Ratio; ESR: Erythrocyte Sedimentation Rate; hsCRP: High-Sensitivity C Reactive Protein; SGOT: Serum Glutamic Oxaloacetic Transaminase; AST: Aspartate Transaminase; SGPT: Serum Glutamate Pyruvate Transaminase; ALT: Alanine Transaminase; ALP: Alkaline Phosphatase; IU: International Unit; TG: Triglycerides; TC: total cholesterol; LDL: Low Density Lipoprotein; HDL: High Density Lipoprotein; VLDL: Very Low Density Lipoprotein; FBG: fasting blood glucose; PPBG: postprandial blood glucose; HOMA-IR: Homeostatic Model Assessment for Insulin Resistance.

**Table 2:** Time domain and frequency domain indices of HRV in the study population.

	Controls	NAFLD without diabetes	NAFLD with diabetes	*p* value
*Time domain indices*				
SDNN (ms)	40.7	30.2	22.2^*∗*^	0.006
	(24.6–61.5)	(15.5–47.8)	(17.59–28.2)	
SDSD (ms)	24.2	25.9	13.5^*∗*#^	0.009
	(17.3–43.2)	(8.8–36.8)	(9.36–19.6)	
pNN50 (%)	0.04	0.04	0.00^*∗*#^	0.008
	(0.00–0.22)	(0.00–0.16)	(0.00–0.01)	
*Frequency domain indices*				
LF nu	40.2	41.9	37.8	0.855
	(33.4–53.6)	(27.1–52.3)	(23.9–53.2)	
HF nu	29.2	26.8	19.7	0.575
	(18.4–43.9)	(15.6–36.3)	(15.5–38.9)	
LF : HF ratio	1.5	1.6	1.6	0.843
	(1.0–2.6)	(1.0–2.2)	(0.9–2.9)	
Total power (ms^2^)	1166.5	658.4	310.6^*∗*^	0.005
	(383.5–2312.6)	(202.9–1379.2)	(173.3–545.4)	

Values are expressed as median (IQR); _ _^*∗*^significant versus control; _ _^#^significant versus NAFLD without diabetes.

SDNN: standard deviation of all NN intervals; SDSD: standard deviation of differences between adjacent NN intervals; pNN50%: NN50 count divided by the total number of all NN intervals; LF: low frequency (0.04–0.15 Hz); HF: high frequency (0.15–0.40 Hz); nu: normalized unit.

**Table 3:** Time domain and frequency domain indices of HRV in controls, Grade I and Grade II NAFLD.

	Controls (*n* = 25)	Grade I (*n* = 31)	Grade II (*n* = 18)	*p* value
Diabetics (*n*, %)	—	16, 51.6%	8, 44.4%	0.62
*Time domain indices*				
SDNN (ms)	40.7	28.3	21.2^*∗*^	0.010
	(24.6–61.5)	(19.5–46.3)	(14.7–26.0)	
SDSD (ms)	24.2	18.8	12.2^*∗*^	0.026
	(17.3–43.2)	(12.4–29.8)	(9.1–28.4)	
pNN50 (%)	0.04	0.01	0.00^*∗*^	0.016
	(0.00–0.22)	(0.00–0.07)	(0.00–0.01)	
*Frequency domain indices*				
LF nu	40.2	37.8	44.5	0.435
	(33.4–53.6)	(27.1–53.3)	(20.1–50.0)	
HF nu	29.2	19.7	23.1	0.650
	(18.4–43.9)	(14.9–37.9)	(19.3–35.8)	
LF : HF ratio	1.5	1.6	1.4	0.804
	(0.9–3.0)	(0.9–3.4)	(0.8–2.7)	
Total power (ms^2^)	1166.5	515.0	255.1^*∗*^	0.006
	(383.5–2312.6)	(271.1–1374.3)	(157.5–382.6)	

Values are expressed as median (IQR); _ _^*∗*^significant versus control.

SDNN: standard deviation of all NN intervals; SDSD: standard deviation of differences between adjacent NN intervals; pNN50%: NN50 count divided by the total number of all NN intervals; LF: low frequency (0.04–0.15 Hz); HF: high frequency (0.15–0.40 Hz); nu: normalized unit.

**Table 4:** Two-way ANOVA for determination of main effect of diabetic status, NAFLD grade, and their interaction.

	Presence of diabetes	NAFLD grade	Interaction between diabetes and NAFLD grade
SDNN	0.054	0.140	0.461
SDSD	0.009^*∗*^	0.817	0.201
Total power	0.001^*∗*^	0.903	0.902

__^*∗*^Significant.

**Table 5:** Linear correlation analysis between the indices of HRV (dependent) and independent variables.

	SDNN		SDSD		Total power	
	*R*	*p* value	*R*	*p* value	*R*	*p* value
Age (yr)	−0.242^*∗*^	0.046	−0.301^*∗*^	0.013	−0.212	0.083
*Variable related to whole body fat*						
BMI (kg/m^2^)	−0.391^*∗*^	0.001	−0.255^*∗*^	0.036	−0.401^*∗*^	0.001
WHR	−0.071	0.563	0.004	0.976	−0.106	0.391
Fat percentage (%)	−0.302^*∗*^	0.012	−0.204	0.095	−0.309^*∗*^	0.010
Fat Mass (kg)	−0.319^*∗*^	0.008	−0.220	0.071	−0.333^*∗*^	0.006
*Variable related to inflammation*						
ESR (mm/hr)	−0.085	0.493	−0.002	0.986	−0.083	0.501
hsCRP (mg/L)	−0.139	0.258	−0.130	0.292	−0.149	0.226
*Variable related to liver function*						
Bilirubin (mg/dL)	−0.033	0.788	−0.067	0.590	−0.026	0.833
SGOT (AST) (IU/L)	0.117	0.344	0.137	0.265	0.062	0.615
SGPT (ALT) (IU/L)	0.007	0.955	0.035	0.779	−0.041	0.739
ALP (IU/L)	−0.137	0.266	−0.103	0.405	−0.137	0.266
*Variable related to lipid profile*						
TG (mg/dL)	−0.240^*∗*^	0.048	−0.178	0.147	−0.281^*∗*^	0.020
TC (mg/dL)	−0.405^*∗*^	0.001	−0.301^*∗*^	0.013	−0.390^*∗*^	0.001
LDL (mg/dL)	−0.359^*∗*^	0.003	−0.286^*∗*^	0.018	−0.333^*∗*^	0.006
HDL (mg/dL)	−0.081	0.512	0.001	0.995	−0.051	0.678
VLDL (mg/dL)	−0.368^*∗*^	0.002	−0.270^*∗*^	0.026	−0.401^*∗*^	0.001
*Variables related to diabetic status*						
FBG (mg/dL)	−0.493^*∗*^	0.000	−0.414^*∗*^	0.000	−0.442^*∗*^	0.000
PPBG (mg/dL)	−0.474^*∗*^	0.000	−0.451^*∗*^	0.000	−0.511^*∗*^	0.000
Fasting insulin (*µ*IU/mL)	−0.097	0.431	−0.020	0.873	−0.088	0.475
HOMA-IR score	−0.225	0.065	−0.150	0.223	−0.200	0.102

__^*∗*^Significant.

BMI: Body Mass Index; WHR: Waist Hip Ratio; ESR: Erythrocyte Sedimentation Rate; hsCRP: High-Sensitivity C Reactive Protein; SGOT: Serum Glutamic Oxaloacetic Transaminase; AST: Aspartate Transaminase; SGPT: Serum Glutamate Pyruvate Transaminase; ALT: Alanine Transaminase; ALP: Alkaline Phosphatase; IU: International Unit; TG: Triglycerides; TC: total cholesterol; LDL: Low Density Lipoprotein; HDL: High Density Lipoprotein; VLDL: Very Low Density Lipoprotein; FBG: fasting blood glucose; PPBG: postprandial blood glucose; HOMA-IR: Homeostatic Model Assessment for Insulin Resistance.

**Table 6:** Stepwise multivariate regression analysis of the indices of HRV with independent variables.

	SDNN		SDSD		Total power	
	Beta	*p* value	Beta	*p* value	Beta	*p* value
Model 1						
TC	−0.482	0.0001	−0.329	0.006	−0.422	0.0001
Model 2						
TC	−0.428	0.0001			−0.369	0.001
Fat%	−0.268	0.014			−0.262	0.020

TC: total cholesterol.

## Abbreviations

ANOVA: Analysis of variance
ALP: Alkaline Phosphatase
ALT: Alanine Transaminase
AST: Aspartate Transaminase
BMI: Body Mass Index
ESR: Erythrocyte Sedimentation Rate
FBG: Fasting blood glucose
FFT: Fast Fourier Transformation
HC: Hip circumference
HDL: High Density Lipoprotein
HF: High frequency (0.15–0.40 Hz)
HOMA-IR: Homeostatic Model Assessment for Insulin Resistance
HRV: Heart rate variability
hsCRP: High-Sensitivity C Reactive Protein
IQR: Interquartile range.

### Reactive Protein

IU: International Unit
LDL: Low Density Lipoprotein
LF: Low frequency (0.04–0.15 Hz)
NAFLD: Nonalcoholic fatty liver disease
nu: Normalized unit
pNN50%: NN50 count divided by the total number of all NN intervals
PPBG: Postprandial blood glucose
PSD: Power spectral density
SDNN: Standard deviation of all NN intervals
SDSD: Standard deviation of differences between adjacent NN intervals
SGOT: Serum Glutamic Oxaloacetic Transaminase
SGPT: Serum Glutamate Pyruvate Transaminase
T2DM: Type 2 diabetes mellitus
TC: Total cholesterol
TG: Triglycerides
VLDL: Very Low Density Lipoprotein
WC: Waist circumference
WHR: Waist Hip Ratio.

## Additional Points

*Strengths and Limitations.* The present study is first study on effect of grade and diabetic status of NAFLD on heart rate variability. The exclusion of subjects with duration longer than 5 years of diabetes provides a suitable group to elucidate the contribution of diabetic status. A limitation of the study is that it is cross-sectional in design with number not sufficient to have subgroup analysis based on Grade I with or without diabetes and Grade II with or without diabetes. A prospective design with long term follow-up can further establish the observation of the present study.

## References

[1] Farrell G. C., Wong V. W.-S., Chitturi S. (2013). NAFLD in Asia—as common and important as in the West. *Nature Reviews Gastroenterology and Hepatology*.

[2] Harrison S. A., Day C. P. (2007). Benefits of lifestyle modification in NAFLD. *Gut*.

[3] Stepanova M., Younossi Z. M. (2012). Independent association between nonalcoholic fatty liver disease and cardiovascular disease in the us population. *Clinical Gastroenterology and Hepatology*.

[4] Hamaguchi M., Kojima T., Takeda N. (2007). Nonalcoholic fatty liver disease is a novel predictor of cardiovascular disease. *World Journal of Gastroenterology*.

[5] Lu H., Zeng L., Liang B., Shu X., Xie D. (2009). High prevalence of coronary heart disease in type 2 diabetic patients with non-alcoholic fatty liver disease. *Archives of Medical Research*.

[6] Cailotto C., Van Heijningen C., Van Der Vliet J. (2008). Daily rhythms in metabolic liver enzymes and plasma glucose require a balance in the autonomic output to the liver. *Endocrinology*.

[7] Sabath E., Báez-Ruiz A., Buijs R. M. (2015). Non-alcoholic fatty liver disease as a consequence of autonomic imbalance and circadian desynchronization. *Obesity Reviews*.

[8] Newton J. L., Pairman J., Wilton K., Jones D. E. J., Day C. (2009). Fatigue and autonomic dysfunction in non-alcoholic fatty liver disease. *Clinical Autonomic Research*.

[9] Sun W., Zhang D., Sun J. (2015). Association between non-alcoholic fatty liver disease and autonomic dysfunction in a Chinese population. *QJM*.

[10] Oni E. T., Agatston A. S., Blaha M. J. (2013). A systematic review: burden and severity of subclinical cardiovascular disease among those with nonalcoholic fatty liver; Should we care?. *Atherosclerosis*.

[11] Thakur M. L., Sharma S., Kumar A. (2012). Nonalcoholic fatty liver disease is associated with subclinical atherosclerosis independent of obesity and metabolic syndrome in Asian Indians. *Atherosclerosis*.

[12] Kaur M., Chandran D., Lal C. (2013). Renal transplantation normalizes baroreflex sensitivity through improvement in central arterial stiffness. *Nephrology Dialysis Transplantation*.

[13] Carthy E. R. (2014). Autonomic dysfunction in essential hypertension: a systematic review. *Annals of Medicine and Surgery*.

[14] Liu Y.-C., Hung C.-S., Wu Y.-W. (2013). Influence of non-alcoholic fatty liver disease on autonomic changes evaluated by the time domain, frequency domain, and symbolic dynamics of heart rate variability. *PLoS ONE*.

[15] Jakovljevic D. G., Hallsworth K., Zalewski P. (2013). Resistance exercise improves autonomic regulation at rest and haemodynamic response to exercise in non-alcoholic fatty liver disease. *Clinical Science*.

[16] Kuehl M., Stevens M. J. (2012). Cardiovascular autonomic neuropathies as complications of diabetes mellitus. *Nature Reviews Endocrinology*.

[17] Hazlehurst J. M., Woods C., Marjot T., Cobbold J. F., Tomlinson J. W. (2016). Non-alcoholic fatty liver disease and diabetes. *Metabolism*.

[18] Souza M. R., Diniz M. D., Medeiros-Filho J. E., Araújo M. S. (2012). Metabolic syndrome and risk factors for non-alcoholic fatty liver disease. *Arquivos de Gastroenterologia*.

[19] Gaggini M., Morelli M., Buzzigoli E., DeFronzo R. A., Bugianesi E., Gastaldelli A. (2013). Non-alcoholic fatty liver disease (NAFLD) and its connection with insulin resistance, dyslipidemia, atherosclerosis and coronary heart disease. *Nutrients*.

[20] Byrne C. D., Targher G. (2015). NAFLD: a multisystem disease. *Journal of Hepatology*.

[21] Nigam P., Bhatt S. P., Misra A., Vaidya M., Dasgupta J., Chadha D. S. (2013). Non-alcoholic fatty liver disease is closely associated with sub-clinical inflammation: a case-control study on Asian Indians in North India. *PLoS ONE*.

[22] Ajmal M. R., Yaccha M., Malik M. A. (2014). Prevalence of nonalcoholic fatty liver disease (NAFLD) in patients of cardiovascular diseases and its association with hs-CRP and TNF-*α*. *Indian Heart Journal*.

[23] Liu Y., Dai M., Bi Y. (2013). Active smoking, passive smoking, and risk of nonalcoholic fatty liver disease (NAFLD): A Population-Based Study in China. *Journal of Epidemiology*.

[24] Oikawa N., Umetsu M., Toyota T., Goto Y. (1986). Quantitative evaluation of diabetic autonomic neuropathy by using heart rate variations: relationship between cardiac parasympathetic or sympathetic damage and clinical conditions. *Tohoku Journal of Experimental Medicine*.

[25] Verrotti A., Loiacono G., Mohn A., Chiarelli F. (2009). New insights in diabetic autonomic neuropathy in children and adolescents. *European Journal of Endocrinology*.

[26] Ueda H., Kuroda N., Ogura M. (1993). Importance of serum cholesterol level in development of diabetic autonomic neuropathy. *Diabetes Research and Clinical Practice*.

[27] Pimenta N. M., Santa-Clara H., Cortez-Pinto H. (2014). Body composition and body fat distribution are related to cardiac autonomic control in non-alcoholic fatty liver disease patients. *European Journal of Clinical Nutrition*.

[28] Targher G., Pichiri I., Zoppini G., Trombetta M., Bonora E. (2012). Increased prevalence of cardiovascular disease in type 1 diabetic patients with non-alcoholic fatty liver disease. *Journal of Endocrinological Investigation*.

[29] Yan L. H., Mu B., Guan Y. (2016). Assessment of the relationship between non-alcoholic fatty liver disease and diabetic complications. *Journal of Diabetes Investigation*.

[30] Idilman I. S., Akata D., Hazirolan T., Doganay Erdogan B., Aytemir K., Karcaaltincaba M. (2015). Nonalcoholic fatty liver disease is associated with significant coronary artery disease in type 2 diabetic patients: A Computed Tomography Angiography Study. *Journal of Diabetes*.

